# Reconstruction of PET Images Using Cross-Entropy and Field of Experts

**DOI:** 10.3390/e21010083

**Published:** 2019-01-18

**Authors:** Jose Mejia, Alberto Ochoa, Boris Mederos

**Affiliations:** 1 Department of Electrical and Computation Engineering, Universidad Autónoma de Ciudad Juárez, Ciudad Juárez 32310, Mexico; 2 Department of Industrial and Systems, Universidad Autónoma de Ciudad Juárez, Ciudad Juárez 32310, Mexico; 3 Department of Physics and Mathematics, Universidad Autónoma de Ciudad Juárez, Ciudad Juárez 32310, Mexico

**Keywords:** positron emission tomography, reconstruction, field of experts

## Abstract

The reconstruction of positron emission tomography data is a difficult task, particularly at low count rates because Poisson noise has a significant influence on the statistical uncertainty of positron emission tomography (PET) measurements. Prior information is frequently used to improve image quality. In this paper, we propose the use of a field of experts to model a priori structure and capture anatomical spatial dependencies of the PET images to address the problems of noise and low count data, which make the reconstruction of the image difficult. We reconstruct PET images by using a modified MXE algorithm, which minimizes a objective function with the cross-entropy as a fidelity term, while the field of expert model is incorporated as a regularizing term. Comparisons with the expectation maximization algorithm and a iterative method with a prior penalizing relative differences showed that the proposed method can lead to accurate estimation of the image, especially with acquisitions at low count rate.

## 1. Introduction

Positron emission tomography is an imaging technology that provides quantitative studies to detect, diagnose, and monitor treatment of different diseases such as hypernated myocardium, cancer, and many others [1,2].

The scan process begins by administering a radioactive substance, the radiotracer, to the patient. The substance is absorbed mainly by the target organs or tissues. The positron emission tomography (PET) data are then obtained by detecting the radiotracer distribution within the body. These acquired data are then processed by reconstruction algorithms to obtain the final image, which is presented to the medical research personnel [2,3].

Quality of the PET image depends of several factors: physical factors such as positron range, non collinearity and spurious events; hardware related factors such as crystal type and size and response time of the electronics; and the software or reconstruction algorithm used to estimate the final image [3].

In this paper, we are interested in improving the quality of the PET images by using algorithms to reconstruct the image. Reconstruction algorithms can be broadly classified into analytic and iterative methods. Iterative methods are popular in PET due to their robustness and ability to incorporate prior data and noise statistics. Iterative methods are mainly based on the maximum likelihood expectation maximization estimator or the least squares model. However, when excessive noise is present in the acquired data, such as in low count acquisitions, most iterative methods have difficulties in obtaining an accurate estimation of the data and regularization techniques are required to stabilize the solution [4].

In this paper a novel approach to reconstruct PET images is presented. Our approach is based on a regularized expectation maximization (EM) algorithm. Here, we propose to regularize teh problem by using a cross-entropy fidelity term and field of experts (FoE) priors [5], which are capable of capturing richer spatial statistics through patches extracted from a dictionary of images. Therefore, we expect that the FoE prior helps to recover the anatomical structure and capture anatomical spatial dependencies of the PET images during the reconstruction process.

The rest of the paper is organized as follows. In Section 2, the proposed algorithm is presented. In Section 3, experiments and results are shown. Finally, conclusions are provided in Section 4.

## 2. Methodology

### 2.1. ML-EM Algorithm

The Maximum Likelihood Expectation Maximization (ML-EM) algorithm takes into account the Poisson-based likelihood distribution of the data.

The PET scanner detectors count pairs of events in coincidence throughout the entire ring. These counts are subsequently processed to form the final image. Here, we represent the counts registered by the *I* detectors of the scanner by the vector $y=[y_{1},y_{2},y_{3},\ldots ,y_{I}]$. Each element $y_{i}$ of *y* is modeled as independent random variables and Poisson distributed with expectation ${\bar{y}}_{i}$ given by

(1) $${\bar{y}}_{i}=E\left[y_{i}\right]=\sum_{j=1}^{J}x_{j}a_{i,j}$$

where $x_{i}$ represents the radionuclide activity within the scanned subject and a pixel in the reconstructed image of size *J* pixels. Here, the image will be ordered as a vector $x=[x_{1},x_{2},x_{3},\ldots ,x_{J}]$. The $a_{i,j}$s are elements of the system matrix *A*. The probability P(y|x) of observing $y_{i}$ is a likelihood of the unknown emissions at pixel $x_{j}$, thus

(2) $$L\left(x\right)=p\left(y|x\right)=\prod_{i=1}^{I}e^{-{\bar{y}}_{i}}\frac{{\bar{y}}_{i}^{y_{i}}}{y_{i}!}$$

The log-likelihood is obtained by combining Equations (1) and (2) as

(3) $$\ln\left(L\left(x\right)\right)=-\sum_{i=1}^{I}\left[\sum_{j=1}^{J}x_{j}a_{i,j}+y_{i}\ln\left(\sum_{j=1}^{J}x_{j}a_{i,j}\right)+\ln\left(y_{i}!\right)\right]$$

The application of maximum-likelihood estimation techniques in Equation (3) leads the EM iterative scheme for the update of the *i*th pixel at iteration (n+1) as follows:

(4) $$x_{j}^{\left(n+1\right)}=\frac{x_{j}^{\left(n\right)}}{\sum_{i}a_{i,j}}\sum_{i}\frac{a_{i,j}y_{i}}{\sum_{k}a_{i,k}x_{k}^{\left(n\right)}}$$

This method has been widely used in PET reconstruction as it produces images with better quality than other techniques. However, it is affected when data are acquired at low-count rates, producing noisy images.

### 2.2. Cross-Entropy

One of the trade offs of the reconstruction algorithms is to minimize the difference between the measured and reconstructed data, while at the same time maintaining the final result without nuisances such as noise. The term that reflects the degree of similarity is often called fidelity term. In this paper, we adopt as fidelity term the cross entropy, which in information theory measures the resemblance between two probability distributions. This approach is taken in [6], where the authors reconstructed the measured data by minimizing a weighted sum of two cross-entropy terms.

The cross-entropy or Kullback–Leiber distance between Ax and *y* is defined as

(5) $$J_{0}\left(x\right)=D\left(y,Ax\right)=\sum_{i}\left(y_{i}\ln y_{i}-y_{i}\ln{\left(Ax\right)}_{i}-y_{i}+{\left(Ax\right)}_{i}\right)$$

It is known that D(y,Ax) is strictly convex and then a sufficient condition for a global minimum is that $\frac{\partial J_{0}\left(x\right)}{\partial x_{j}}=0$, due to

(6) $$\frac{\partial J_{0}\left(x\right)}{\partial x_{j}}=\frac{\partial D(y,Ax)}{\partial x_{j}}=-\frac{a_{i,j}y_{i}}{q_{i}^{\left(n\right)}}+a_{i,j},$$

and then the sufficient condition is written as

(7) $$-\frac{a_{i,j}y_{i}}{q_{i}^{\left(n\right)}}+a_{i,j}=0.$$

It is possible to develop an optimization method based on the EM scheme. In [6], it has been shown that the minimization of Equation (5) with respect to *x* is equivalent to the maximization of the log-likelihood function in the ML estimate of Equation (4). Thus, using the EM algorithm to minimize Equation (5), and using $q_{i}^{\left(n\right)}=\sum_{k}a_{i,k}x_{k}^{\left(n\right)}$ and Equation (6), we have

(8) $$x_{j}^{\left(n+1\right)}=\frac{x_{j}^{\left(n\right)}}{\sum_{i}a_{i,j}y_{i}}\sum_{i}\frac{a_{i,j}y_{i}}{q_{i}^{\left(n\right)}}$$

(9) $$x_{j}^{\left(n+1\right)}=\frac{x_{j}^{\left(n\right)}}{\sum_{i}a_{i,j}}\sum_{i}\frac{a_{i,j}\left(y_{i}+q_{i}^{\left(n\right)}-q_{i}^{\left(n\right)}\right)}{q_{i}^{\left(n\right)}}$$

(10) $$x_{j}^{\left(n+1\right)}=x_{j}^{\left(n\right)}-\frac{x_{j}^{\left(n\right)}}{\sum_{i}a_{i,j}}\sum_{i}\left(-\frac{a_{i,j}y_{i}}{q_{i}^{\left(n\right)}}+a_{i,j}\right)$$

Now, using Equation (6), we obtain

(11) $$x_{j}^{\left(n+1\right)}=x_{j}^{\left(n\right)}-\frac{x_{j}^{\left(n\right)}}{\sum_{i}a_{i,j}}\frac{\partial J_{0}\left(x^{\left(n\right)}\right)}{\partial x_{j}}$$

This scheme is termed as MXE1 in [6] and has been used in several algorithms for reconstruct PET images [7,8]. It worth to remarking that Equation (11) can be seem as a gradient descent step of the cross-entropy function $J_{0}$ with variable step-size $\frac{x_{j}^{\left(n\right)}}{\sum_{i}a_{i,j}}$. Additionally, in this context, this idea can accommodate more complex objective functions, as will be shown in Section 2.4.

### 2.3. Field of Experts Model

The Field-of-expert scheme [5,9] models high-dimensional probability distributions by taking the product of several distributions (the experts). This provides a framework for learning image priors from sets of experts. Each expert distribution takes into account certain image structure learned using a database of images. In this manner, a prior captures richer spatial statistics present in the image. The modeled priors represent clique potential functions on a Markov random field (MRF) [10], and the experts are modeled as t-distributions, defined on each clique, with parameters learned by using the contrastive divergence method of Carreira-Perpinan and Hinton [11]. Field of expert have been used in several image processing algorithms and applications, such as segmentation [12], painting [13], and compressed sensing based restoration [14], among others.

In this paper, we propose to model the prior distribution for PET images by using a field of experts. Each expert is represented as a distribution modeled by linear filters *J*. Thus, the prior function is defined as

(12) $$P\left(x,\Theta \right)=\frac{1}{Z(\Theta )}\prod_{k=1}^{K}\prod_{i=1}^{N}\phi \left(J_{i}^{T}x_{\left(k\right)},\alpha_{i}\right)$$

where $\Theta =\theta_{1},\ldots ,\theta_{N}$ is a set of learned parameters $\theta_{i}=\left\{\alpha_{i},J_{i}\right\}$, $\alpha_{i}$ is a parameter of expert *i*, Z(Θ) is the normalizing function, and ϕ(·,·) represents the experts, and are given by the student t-experts

(13) $$\phi \left(J_{i}^{T}x,\alpha_{i}\right)={\left(1+\frac{1}{2}{\left(J_{i}^{T}x\right)}^{2}\right)}^{-\alpha_{i}})$$

Note that α is similar to the degrees of freedom [15].

Several techniques have been developed to take into account the similarity between the anatomical and functional images to define priors to improve the reconstruction process [16,17].

**Figure 1:**
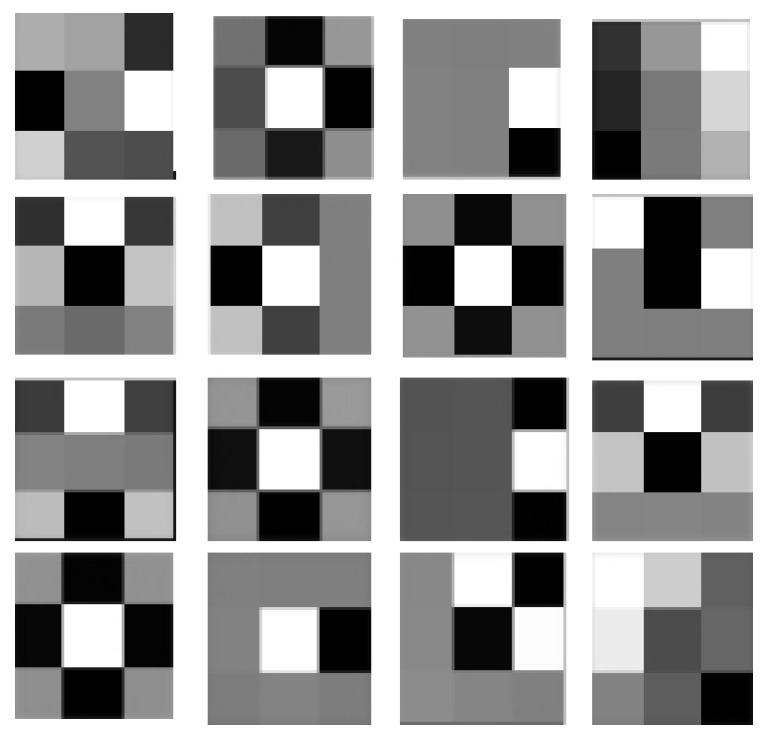
The 5×5 filters obtained by training the oroduct-of-experts model on positron emission tomography (PET) images database. The colors in each frame are proportional to the magnitude of the filter coefficient, using a gray scale.

In this work, to capture anatomical spatial dependencies of the PET images, we use the FoE scheme. To this end, we trained 5×5 filters to model priors that adapt to the images and underlaying anatomy. The training data consisted of 1000 images of real and simulated PET images. We also included a set of 50 images of studies of computer tomography and magnetic resonance without PET to provide the filter with more detailed anatomical characteristics. The obtained filter are shown in Figure 1.

In this way, we could incorporate into the reconstruction filters adapted to structure of the PET images and anatomical information, in the form of organ and lesion boundaries, derived from CT and MR.

### 2.4. Proposed Objective Function

In this section, we present our proposed function:

(14) $$J_{FoE}\left(x\right)=D\left(y,Ax\right)+\beta P\left(x,\Theta \right)$$

where *D* is the cross entropy and *P* is a prior based in the FoE framework. To derive an optimization method, we examine the derivative of Equation (14).

(15) $$\begin{array}{cc} \frac{\partial J_{FoE}\left(x\right)}{\partial x_{j}} & =\frac{\partial D(y,Ax)}{\partial x_{j}}+\beta \frac{\partial P(x,\Theta )}{\partial x_{j}}\\ & =\sum_{i}\left[\frac{-y_{i}a_{i,j}}{{\left(Ax\right)}_{i}}+a_{i,j}\right]+\beta \sum_{k}^{N}J_{-}^{\left(k\right)}*\phi^{\prime }\left(J^{\left(k\right)}*x;\alpha_{i}\right), \end{array}$$

where $J^{\left(k\right)}$ is the convolutional filter corresponding to $J_{k}$ and $J_{-}^{\left(k\right)}$ is obtained by mirroring $J^{\left(k\right)}$ around the center (for more details, see [5]).

Then, we can generalize the idea of (MXE1 iterative scheme) to the proposed regularization of the cross-entropy (Equation (14))

(16) $$x_{j}^{\left(n+1\right)}=x_{j}^{\left(n\right)}-\frac{x_{j}^{\left(n\right)}}{\sum_{i}a_{i,j}}\frac{\partial J_{FoE}\left(x^{\left(n\right)}\right)}{\partial x_{j}}$$

This iterative scheme corresponds to a gradient descent step of the proposed functional $J_{FoE}$ with variable step-size $\frac{x_{j}^{\left(n\right)}}{\sum_{i}a_{i,j}}$, as mentioned in Section 2.2.

Unfortunately, MXE1 offer no guarantee that the algorithm will preserve non-negativity constraints. This can be remedied by using the line search algorithm LINU described in [18] by setting all negative elements in the new iteration to 0. For implementation of the algorithm, we used a fixed iteration scheme, with 27 iterations, and a β=0.5.

## 3. Results

In this section, we present acquired and simulated datasets of PET images to show how the proposed algorithm deals with noise and structures and borders. We also offer comparisons with the EM algorithm and the algorithm proposed in [19], which uses a concave prior penalizing relative differences (CP) between neighbors. This algorithm was modified in [20] (GE healthcare white paper). Here, we set the parameters of the CP algorithm as β=0.01, γ=0.1, and λ=0.97. The EM was used with 12 iterations.

The simulated images were generated using Simset (a Simulation System for Emission Tomography) software (version 2.9.2, provided by the Division of Nuclear Medicine, University of Washington, Seattle, WA, USA) [21]. We used a simulated 3D PET detector with two axial rings consisting of an aluminum front cover followed by a single layer of 3.5 cm of BGO. From the generated volumetric data, 2D sinograms were taken.

### Simulated Data

In this experiment, the efficacy of the reconstruction under low count conditions was evaluated. We used the reconstructed images from each method to generate selected surfaces to show graphically how close the reconstructed image was to the ground truth.

**Figure 2:**
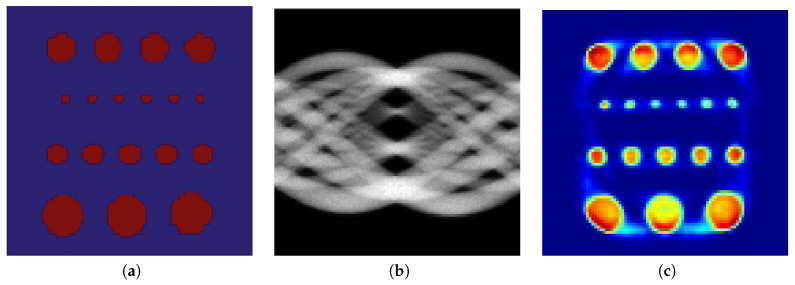
Cylindrical software phantom: (**a**) ground truth; (**b**) simulated sinogram data at 30 M counts; and (**c**) reconstruction with expectation maximization (EM).

To this end, we designed a cylindrical software phantom of polymethyl methacrylate with five rows of holes (water-filled cylindrical inserts) of 2, 3, 4, and 5 mm in diameter. Each hole was filled with activity of 1:8 with respect to background. Figure 2 shows the phantom, a simulation of 30 M counts and its reconstruction using expectation maximization (EM).

**Figure 3:**
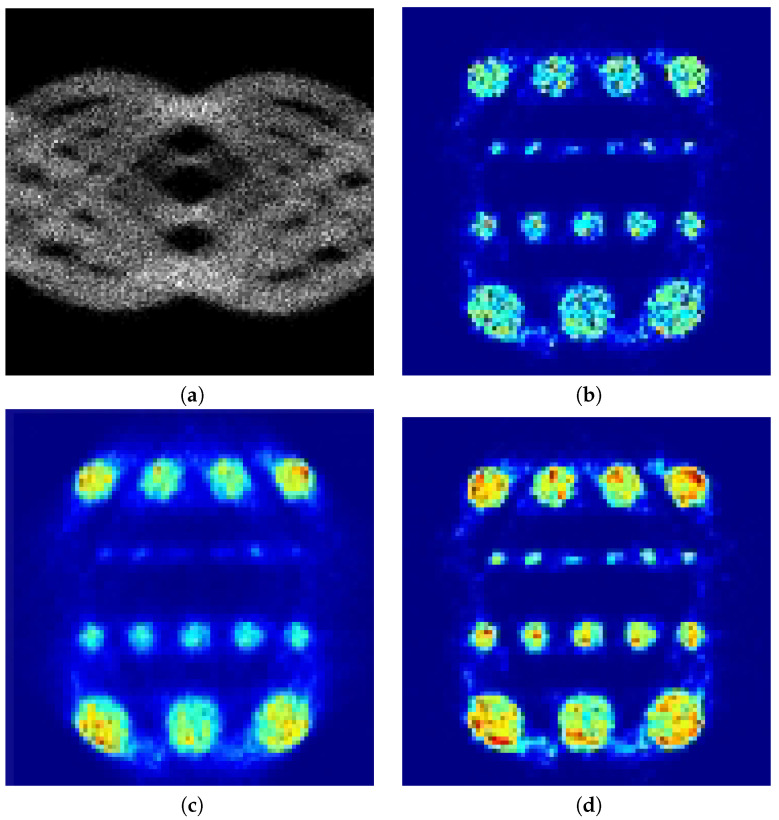
Cylindrical software phantom: (**a**) Input sinogram; (**b**) low count reconstruction with EM; (**c**) low count reconstruction with CP; and (**d**) low count reconstruction with the proposed method.

We were interested in low count reconstructions, since this means less radiation exposure for the patient. Similar to higher counts data, all methods evaluated had practically equal quality in the reconstruction. Thus, we ran a PET scan simulation at 5 M count using the Simset software. Figure 3a shows the simulated sinogram data at lower count and Figure 3b–d shows the reconstruction using EM, CP, and the proposed method, respectively. It can be seen that the image reconstructed with the proposed method had better definition of the rods.

**Figure 4:**
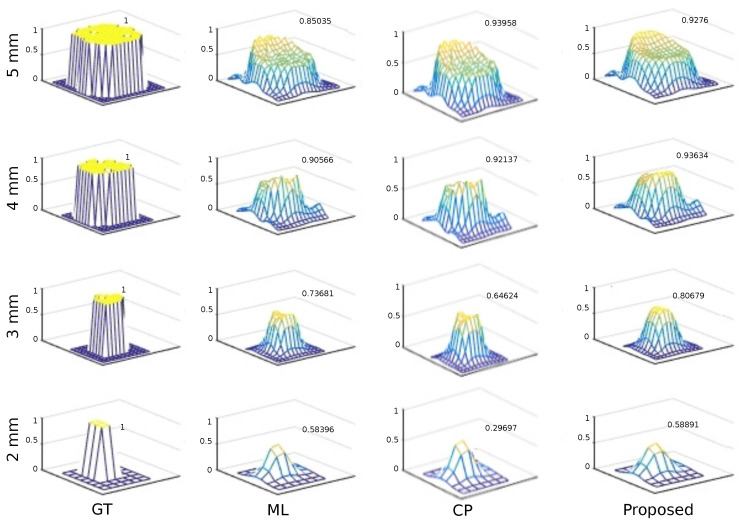
Profiles of the different methods with the cylindrical software phantom. Each row shows the same hole, and each column the same method. The maximum of each surface is indicated next to it.

Figure 4 depicts the surfaces for each row of the holes. The proposed method had a smoother surface than EM, without losing contrast. In addition, contrast for the larger cylinders was slightly better at some points in the surface with CP, and achieved a maximum for the 5 mm surface.

In our next experiment, we used the Digimouse anatomical atlas dataset described in [22] to design a software mouse phantom. The mouse phantom was used to have a more realistic assessment of the structures found on practical data. The PET scans of the mouse were simulated using Simset software (version, publisher, city, state abbreviation if USA or Canada, country).

**Figure 5:**
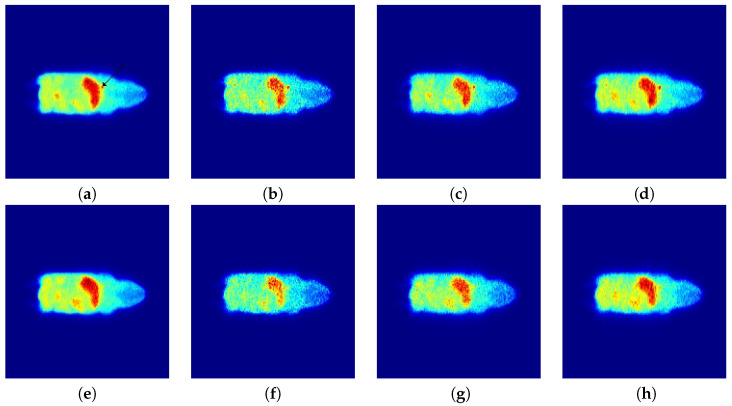
Slices of the Digimouse software phantom: ground truth (**a**,**e**); low count reconstruction with EM (**b**,**f**); low count reconstruction with CP (**c**,**g**); and (**d**,**h**) low count reconstruction with the proposed method. In (**a**), the arrow indicates a lesion.

We simulated two realizations with different number of counts: 30 M and 5 M counts. The 30 M counts realization was taken as a ground truth. Figure 5 presents two different slices of the phantom, the first column shows the 30 M realization reconstructed with EM, while the second, third, and fourth columns show the 5 M realization reconstructed with EM, CP and the proposed method, respectively.

**Figure 6:**
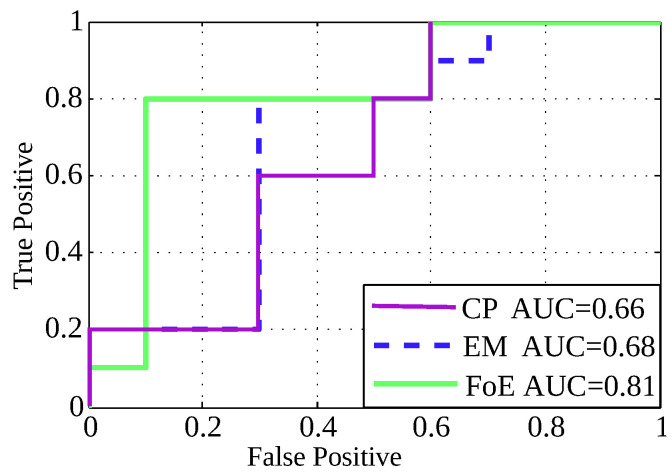
ROC analysis evaluated on the Digimouse phantom.

To quantitatively evaluate image quality in the Digimouse, a channelized Hoteling observer [23,24] was used in the context of lesion detectability. The area under the curve (AUC) was used as a figure of merit. The task performed by the observer was the detection of a lesion with known location. To this end, we used 25 simulations with lesion and 25 without lesion. The lesion has an activity of 1:5 with respect to background, in 10 of the 25 phantoms with lesion, and 1:3 with respect to background in the rest. The images were reconstructed with each method. These images were fed to the observer to analyze its output. Figure 6 shows the AUC attained by each method. As can be seen, our method obtained more AUC than EM and CP. Based on this result, the simulated observer was able to better detect the lesion in the images reconstructed by the proposed method.

**Figure 7:**
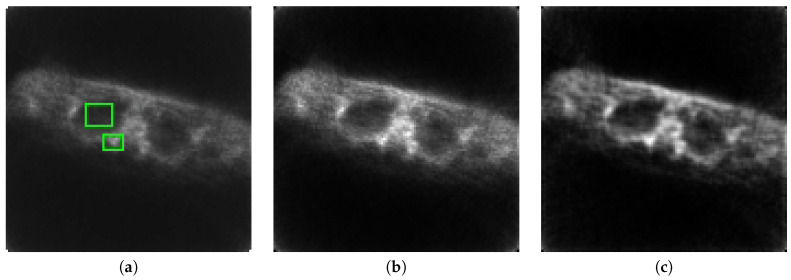
Reconstruction of measured data with: (**a**) EM; (**b**) CP; and (**c**) the proposed method.

In the next experiment, we evaluated the performance with measured data. We used data from http:\\web.eecs.umich.edu\~fessler\, which is from a subject who was scanned on a CTI ECAT PET scanner. The raw sinograms were acquired at 160 radial samples and 192 angular samples; data were pre-corrected for delayed coincidences [25]. Figure 7a–c shows the reconstructions of the data using EM, CP, and the proposed method, respectively.

Table 1 shows the results of applying the contrast resolution (CR) metric [18] between the two rectangles depicted in Figure 7a. Our method has better CR than the other methods evaluated, and lower noise.

## 4. Conclusions

In this paper, a novel reconstruction method for PET images is presented based on a cross-entropy fidelity term. We propose to regularize the ill-posed problem by using field of experts priors. In this way, we can incorporate into the reconstruction process prior distributions from adapted filters to structure PET images and anatomical information.

The experimental results show that the proposed method led to a better reconstruction performance than EM and CP. In an experiment with a phantom with rods of different sizes, we found an improvement in the recovering of the pixels of the smallest rods, showing that the proposed method could perform better in the reconstruction of small structures such as lesions. This was also observed in a second experiment with a phantom with lesions, where a ROC analysis of a observer detecting lesions was simulated and the proposed method outperformed EM and CP methods. In the experiment with measured data, our method obtained more contrast resolution, and visually the image of the proposed method had more defined edges than EM and CP. As future work, we plan to implement the algorithm with listmode data and take into account the additional information of time of flight, as well as construct a database of more images including PET/CT and PET/MRI.

## Figures and Tables

**Table 1:** Contrast resolution measures.

Method	CR
EM	0.577
CP	0.541
Proposed	0.695
